# Atractylodes macrocephala-Paeonia lactiflora Class Formula for the Treatment of Irritable Bowel Syndrome: A Systematic Review With Meta-Analysis and Trial Sequential Analysis

**DOI:** 10.7759/cureus.49997

**Published:** 2023-12-05

**Authors:** Wenjun Bai, Zhe Wang, Junwei Liang, Hualiang Deng

**Affiliations:** 1 Department of Acupuncture, Affiliated Hospital of Shandong University of Traditional Chinese Medicine, Jinan, CHN; 2 Department of Gastroenterology, The First Clinical Medical College, Shandong University of Traditional Chinese Medicine, Jinan, CHN; 3 Department of Acupuncture and Massage, Shandong University of Traditional Chinese Medicine, Jinan, CHN; 4 Department of Gastroenterology II, Affiliated Hospital of Shandong University of Traditional Chinese Medicine, Jinan, CHN

**Keywords:** trial sequential analysis, meta-analysis, irritable bowel syndrome, paeonia lactiflora, atractylodes macrocephala

## Abstract

Previous meta-analyses suggested that Chinese herbal medicine (CHM) is effective for irritable bowel syndrome (IBS). Formulas with *Atractylodes macrocephala* and *Paeonia lactiflora* as the core pairs have been widely used by traditional Chinese medicine (TCM) practitioners for the treatment of IBS. We aimed to examine the efficacy and safety of the *Atractylodes macrocephala*-*Paeonia lactiflora* class formula (A-P CHM) for IBS through a meta-analysis and trial-sequential analysis (TSA). The protocol is registered in the International Prospective Register of Systematic Reviews (PROSPERO) under registration number CRD42023439087. We searched seven databases for data up to May 23, 2023. The primary outcome was global IBS symptom relief. The secondary outcomes included the IBS severity scoring system (IBS-SSS) score and treatment-related adverse events. The relative ratio (RR) (dichotomous variables), the standardized mean difference (SMD) (continuous variables), the number needed to treat (NNT), the number needed to harm (NNH), and the required information size (RIS) were calculated. Twenty-four eligible articles with 3,768 participants were included. Thirteen trials were at low risk of bias (RoB). Compared with placebo or Western medication, A-P CHM was associated with a significantly higher proportion of relief of global IBS symptoms. The TSA analysis verified the primary outcome. For the secondary outcome, the A-P CHM IBS-SSS score was lower than Western medication or placebo at the end of the treatment, which was further confirmed by the TSA analysis. We asserted that A-P CHM might be a potential candidate for patients with IBS, especially for IBS-D. It may provide a theoretical basis for future optimization of irritable bowel syndrome with diarrhea (IBS-D) herbal formulas. The overall certainty of the evidence was not high; more tightly designed randomized controlled trials (RCTs) are required in the future.

## Introduction and background

Irritable bowel syndrome (IBS) is a chronic functional bowel disorder characterized by abdominal pain linked to disordered defecation [1]. Affecting 5%-10% of the global population, IBS is notably more prevalent in women and younger individuals [2]. Although the pathogenesis of IBS remains poorly understood, the prevailing scholarly consensus posits abnormal regulation of the brain-gut axis (BGA) resulting from a complex interplay of factors. These include increased visceral sensitivity, gastrointestinal motility disorders, low-grade intestinal inflammation, gut microflora imbalance, psychosomatic stress, and immune activation [3]. Many treatments are proposed for IBS [4-6], and dietary interventions and pharmacological treatments are usually the primary choices [5, 7]. Due to the complex pathophysiology of IBS, dietary interventions and pharmacological treatments are only partially effective, yielding marginal improvements in comparison to placebo [7, 8]. In instances where conventional pharmacological treatments fail to alleviate IBS symptoms, patients often resort to complementary and alternative medicines (CAMs) such as herbal medicine and acupuncture [9].

According to Chinese medicine (CM) theory, the occurrence of IBS is related to emotional disorders, over-conscientiousness, and eating disorders. The primary pathogenesis encompasses gān (liver) depression, pí (spleen) deficiency, and an overabundance of dampness. In a CM clinic, the treatment methods mainly focus on promoting the qi flow of gān, improving emotional depression, strengthening pí, eliminating dampness, and alleviating pain and other symptoms [10]. Nearly 50% of IBS patients have sought herbal medications as an alternative therapy at least once in their lifetime [11-13], and the use of herbal therapy is still growing [14], especially when conventional medications are ineffective in relieving IBS symptoms.

Formulas featuring *Atractylodes macrocephala *and *Paeonia lactiflora* as key components are widely employed by traditional Chinese medicine (TCM) practitioners for addressing disorders such as diarrhea, intestinal tinnitus, and spleen deficiency. Analysis of the TCM application data from the Chinese Formulas Database revealed a total of 1,557 formulas incorporating *Atractylodes macrocephala* and *Paeonia lactiflora* pairs [15, 16]. Modern clinics similarly utilize compound formulas with *Atractylodes macrocephala *and *Paeonia lactiflora* as the core pair for treating irritable bowel syndrome, demonstrating substantial clinical efficacy [17]. *Atractylodes macrocephala* and *Paeonia lactiflora* were first published in Shennong Ben Cao Jing (Divine Husbandman's Classic of the Materia Medica). *Atractylodes macrocephala* is sweet, bitter, warm, and bitter-warm to dry dampness and strengthen the spleen and stop diarrhea; *Paeonia lactiflora* is bitter, sour, slightly cold, and sour-cold to drain fire, suppress wood, and pacify the liver [18]. A pertinent study indicated that the *Atractylodes Macrocephala*-*Paeonia lactiflora* (A-P CHM) pair surpassed *Atractylodes Macrocephala* or *Paeonia lactiflora* alone in the treatment of IBS by more effectively reducing the levels of 5-hydroxytryptamine, vasoactive intestinal peptide, and growth inhibitors, stabilizing mast cells, diminishing intestinal hypersensitivity, and enhancing fecal water content in IBS rats [19].

While prior studies have conducted meta-analyses of herbal remedies for IBS [20], there has been no systematic evaluation or meta-analysis of the A-P CHM for IBS to date. Given this context, we undertook a meta-analysis and systematic evaluation using trial sequential analysis (TSA) of the A-P CHM for IBS. Our objective was to scrutinize the therapeutic efficacy of A-P CHM for IBS and to investigate the necessity for new trials targeting specific outcomes, such as the relief of IBS symptoms.

## Review

Methods

Search Strategy and Eligibility Criteria

The protocol is registered in the International Prospective Register of Systematic Reviews (PROSPERO) (registration number: CRD42023439087). We searched seven databases: PubMed, Excerpta Medica database (Embase), Ovid Medical Literature Analysis and Retrieval System Online (MEDLINE), the Cochrane Library, SinoMed, the Chinese National Knowledge Infrastructure Databases (CNKI), and Wanfang Database, for data up to May 23, 2023. There were no language restrictions. The subject/medical subject headings (MeSH) terms used for the searches were "*Atractylodes macrocephala* Koidz" OR "*Radix Paeoniae Alba*" OR "medicine, Chinese traditional" combined with "irritable bowel syndrome". Search strategies are shown in the supplementary files (S1-4 Table). Irritable bowel syndrome should be diagnosed according to the Rome criteria or the criteria recommended in the guidelines. In randomized controlled trials (RCTs) examining the effect of herbal medicine (including *Atractylodes macrocephala* and *Paeonia lactiflora*) therapies in adult patients (≥18 years) with IBS, the control arms were required to receive a placebo, Western medication, or a physician’s “usual management”. At least one of the following outcomes had to be presented for evaluation by the included trial: relief of global IBS symptoms; adequate relief response rate; the multiple endpoints recommended by the US Food and Drug Administration for IBS; the IBS severity scoring system (IBS-SSS); duration of therapy: ≥ 4 weeks. We used the time point at the end of the treatment.

The search strategy and search terms used are summarized in Tables 1-4.

**Table 1:** PubMed search history

Search number	Query	Sort by	Filters	Search details	Results
3	("Irritable Bowel Syndrome" or "Irritable Bowel Syndromes" or " Syndrome, Irritable Bowel" or " Syndromes, Irritable Bowel" or "Colon, Irritable" or "Irritable Colon" or "Colitis, Mucous" or "Colitides, Mucous" or " Mucous Colitis") AND ("Medicine, Chinese Traditional" or "Zhong Yi Xue" or "Chinese Medicine, Traditional" or "Chinese Traditional Medicine" or "Traditional Chinese Medicine" or "Traditional Medicine, Chinese" or "Drugs, Chinese Herbal" or "Chinese Herbal Drugs" or "Chinese Plant Extracts" or "Baizhu" or "Atractylodes macrocephala Koidz" or "Largehead Atractylodes Rh" or "Rhizoma Atractylodis Macrocephalae" or "Baishao" or "Cynanchum otophyllum" or "White Paeony Root" or "Paeoniae Radix Alba" or "Radix Paeoniae Alba")			("Irritable Bowel Syndrome"[All Fields] OR "Irritable Bowel Syndromes"[All Fields] OR "syndrome irritable bowel"[All Fields] OR "syndromes irritable bowel"[All Fields] OR "colon irritable"[All Fields] OR "Irritable Colon"[All Fields] OR "colitis mucous"[All Fields] OR "colitides mucous"[All Fields] OR "Mucous Colitis"[All Fields]) AND ("medicine chinese traditional"[All Fields] OR "Zhong Yi Xue"[All Fields] OR "chinese medicine traditional"[All Fields] OR "Chinese Traditional Medicine"[All Fields] OR "Traditional Chinese Medicine"[All Fields] OR "traditional medicine chinese"[All Fields] OR "drugs chinese herbal"[All Fields] OR "Chinese Herbal Drugs"[All Fields] OR "Chinese Plant Extracts"[All Fields] OR "Baizhu"[All Fields] OR "Atractylodes macrocephala Koidz"[All Fields] OR ("largehead"[All Fields] AND ("atractylodes"[MeSH Terms] OR "atractylodes"[All Fields] OR "atractylode"[All Fields]) AND ("rehabilitation"[MeSH Subheading] OR "rehabilitation"[All Fields] OR "rh"[All Fields])) OR "Rhizoma Atractylodis Macrocephalae"[All Fields] OR "Baishao"[All Fields] OR "Cynanchum otophyllum"[All Fields] OR "White Paeony Root"[All Fields] OR "Paeoniae Radix Alba"[All Fields] OR "Radix Paeoniae Alba"[All Fields])	413
2	"Irritable Bowel Syndrome" or "Irritable Bowel Syndromes" or " Syndrome, Irritable Bowel" or " Syndromes, Irritable Bowel" or "Colon, Irritable" or "Irritable Colon" or "Colitis, Mucous" or "Colitides, Mucous" or " Mucous Colitis"			"Irritable Bowel Syndrome"[All Fields] OR "Irritable Bowel Syndromes"[All Fields] OR "syndrome irritable bowel"[All Fields] OR "syndromes irritable bowel"[All Fields] OR "colon irritable"[All Fields] OR "Irritable Colon"[All Fields] OR "colitis mucous"[All Fields] OR "colitides mucous"[All Fields] OR "Mucous Colitis"[All Fields]	17,817
1	"Medicine, Chinese Traditional" or "Zhong Yi Xue" or "Chinese Medicine, Traditional" or "Chinese Traditional Medicine" or "Traditional Chinese Medicine" or "Traditional Medicine, Chinese" or "Drugs, Chinese Herbal" or "Chinese Herbal Drugs" or "Chinese Plant Extracts" or "Baizhu" or "Atractylodes macrocephala Koidz" or "Largehead Atractylodes Rh" or "Rhizoma Atractylodis Macrocephalae" or "Baishao" or "Cynanchum otophyllum" or "White Paeony Root" or "Paeoniae Radix Alba" or "Radix Paeoniae Alba"			"medicine chinese traditional"[All Fields] OR "Zhong Yi Xue"[All Fields] OR "chinese medicine traditional"[All Fields] OR "Chinese Traditional Medicine"[All Fields] OR "Traditional Chinese Medicine"[All Fields] OR "traditional medicine chinese"[All Fields] OR "drugs chinese herbal"[All Fields] OR "Chinese Herbal Drugs"[All Fields] OR "Chinese Plant Extracts"[All Fields] OR "Baizhu"[All Fields] OR "Atractylodes macrocephala Koidz"[All Fields] OR ("largehead"[All Fields] AND ("atractylodes"[MeSH Terms] OR "atractylodes"[All Fields] OR "atractylode"[All Fields]) AND ("rehabilitation"[MeSH Subheading] OR "rehabilitation"[All Fields] OR "rh"[All Fields])) OR "Rhizoma Atractylodis Macrocephalae"[All Fields] OR "Baishao"[All Fields] OR "Cynanchum otophyllum"[All Fields] OR "White Paeony Root"[All Fields] OR "Paeoniae Radix Alba"[All Fields] OR "Radix Paeoniae Alba"[All Fields]	142,564

**Table 2:** Embase search history Embase: Excerpta Medica database

No. and query	Results
#3. #1 AND #2	1,057
#2. 'irritable bowel syndrome' OR 'irritable bowel syndromes' OR 'syndrome, irritable bowel' OR 'syndromes, irritable bowel' OR 'colon,irritable' OR 'irritable colon' OR 'colitis,mucous' OR 'colitides, mucous' OR 'mucous colitis'	35,276
#1. 'medicine, chinese traditional'/exp OR 'medicine,chinese traditional' OR 'zhong yi xue' OR 'chinese medicine, traditional' OR 'chinese traditional medicine'/exp OR 'chinese traditional medicine' OR 'traditional chinese medicine'/exp OR 'traditional chinese medicine' OR 'traditional medicine, chinese' OR 'drugs, chinese herbal'/exp OR 'drugs, chinese herbal' OR 'chinese herbal drugs' OR 'chinese plant extracts' OR baizhu'/exp OR 'baizhu' OR 'atractylodes macrocephala koidz'/exp OR 'atractylodes macrocephala koidz' OR 'largehead atractylodesrh' OR 'rhizoma atractylodis macrocephalae'/exp OR 'rhizoma atractylodis macrocephalae' OR baishao'/exp OR 'baishao' OR 'cynanchum otophyllum'/exp OR 'cynanchum otophyllum' OR 'white paeony root' OR 'paeoniae radix alba'/exp OR 'paeoniae radix alba' OR 'radix paeoniae alba'/exp OR 'radix paeoniae alba'	212,585

**Table 3:** Ovid MEDLINE Search History MEDLINE: Medical Literature Analysis and Retrieval System Online

#	Query	Results from 25 May 2023
1	Irritable Bowel Syndrome/	9,388
2	("Irritable Bowel Syndromes" or " Syndrome, Irritable Bowel" or " Syndromes, Irritable Bowel" or "Colon, Irritable" or "Irritable Colon" or "Colitis, Mucous" or "Colitides, Mucous" or " Mucous Colitis").mp. [mp=title, book title, abstract, original title, name of substance word, subject heading word, floating sub-heading word, keyword heading word, organism supplementary concept word, protocol supplementary concept word, rare disease supplementary concept word, unique identifier, synonyms, population supplementary concept word, anatomy supplementary concept word]	602
3	1 or 2	9,830
4	exp Medicine, Chinese Traditional/	23,658
5	exp Drugs, Chinese Herbal/	51,931
6	exp Atractylodes/	485
7	exp Paeonia/	1,438
8	4 or 5 or 6 or 7	69,023
9	3 and 8	148

**Table 4:** Cochrane Library search history

ID	Search
#1	(Medicine, Chinese Traditional):ti,ab,kw OR (Zhong Yi Xue):ti,ab,kw OR (Chinese Medicine, Traditional):ti,ab,kw OR (Chinese Traditional Medicine):ti,ab,kw OR (Traditional Chinese Medicine):ti,ab,kw (Word variations have been searched)
#2	(Traditional Medicine, Chinese):ti,ab,kw OR (Drugs, Chinese Herbal):ti,ab,kw OR (Chinese Herbal Drugs):ti,ab,kw OR (Chinese Plant Extracts):ti,ab,kw (Word variations have been searched)
#3	(Baizhu):ti,ab,kw OR (Atractylodes macrocephala Koidz):ti,ab,kw OR (Largehead Atractylodes Rh):ti,ab,kw OR (Rhizoma Atractylodis Macrocephalae):ti,ab,kw (Word variations have been searched)
#4	(Baishao):ti,ab,kw OR (Cynanchum otophyllum):ti,ab,kw OR (White Paeony Root):ti,ab,kw OR (Paeoniae Radix Alba):ti,ab,kw OR (Radix Paeoniae Alba):ti,ab,kw (Word variations have been searched)
#5	#1 or #2 or #3 or #4

Exclusion Criteria

We excluded studies on Chinese medicine combined with other non-*Atractylodes macrocephala*-*Paeonia lactiflora* prescriptions, studies where the control group was treated with traditional Chinese medicine, and studies where the full text of the publication could not be obtained. We also excluded studies that were conference articles and not RCTs.

Outcome Assessment

The primary outcomes assessed were the relief of global IBS symptoms, which were defined by the following: (1) the recent consensus that patients self-reported relief of symptoms; (2) an IBS-SSS score＜75 points or conditions improved by one level after the last treatment, compared with before treatment; (3) adopting the United States Food and Drug Administration (FDA) criteria for IBS remission response to both abdominal pain and stool consistency; (4) an adequate relief response rate.

The secondary outcomes included the IBS-SSS score and treatment-related adverse events.

Data Abstraction

Data were extracted independently by WJ B and Z W using standardized extraction forms. Data were extracted as intention-to-treat analyses, with dropouts assumed to be treatment failures, whenever trial reporting allowed this, in accordance with the guidance provided by the Cochrane Handbook for Systematic Reviews of Interventions [21].

Risk of Bias Assessment

The Cochrane Risk of Bias (RoB) tool (RoB 2.0) (https://www.riskofbias.info) [22] was used to assess the risk of bias at the individual study level, which was independently done by JW L and Z W. Each domain of a trial was rated with low, high, or some concerns, and an overall RoB was lastly rated for the trial. discrepancy in the RoB assessment was solved by discussion.

Data Analysis

The data were analyzed using the R software (version 4.1.1, The R Core Team, R Foundation for Statistical Computing, Vienna, Austria). Separate analyses were performed to compare the efficacy and safety of the A-P CHM in all studies compared with placebo or other drugs for the treatment of IBS (e.g., improvement in global symptoms of IBS, IBS-SSS, composite FDA endpoint, FDA endpoint abdominal pain relievers, and FDA endpoint intestinal symptom relievers).

For dichotomous variables, relative ratios (RR) and their corresponding 95% confidence intervals (CIs) were calculated; for continuous variables, mean scores and SD were extracted using a standardized mean difference (SMD) with 95% CIs. Data were presented in forest plots. We planned to assess for evidence of publication bias by applying Egger’s test (for which p<0.05 is significant to suggest publication bias) to funnel plots of odds ratios (ORs) [23] for 10 studies or more [24].

Heterogeneity was evaluated using the I² statistic (low heterogeneity I²<25%, moderate I²=25-50%, and high I²>50%), and homogeneity was evaluated using the Q statistic (with p<0·10 considered statistically significant) [25]. A p-value＜0.05 was considered statistically significant.

The number needed to treat (NNT) and the number needed to harm (NNH) were calculated using the formula NNT or NNH = 1/(control event rate - experimental event rate).

The TSA was performed by TSA 0.9.5.10 beta (https://www.ctu.dk/tsa/) to calculate the required information size (RIS). The type I error was allowed to be 0.05, and the type II error was 0.2 when estimating the RIS. The significance boundaries were calculated based on the O’Brien-Fleming alpha-spending method. For continuous data, we estimated the mean difference and variance based on empirical assumptions generated by software. For dichotomous data, we estimated the mean difference and variance based on the incidence of low risk of bias studies.

Certainty Assessment

The GRADEprofiler (gradeworkinggroup.org) was used to evaluate the overall certainty of evidence across RCTs, which was independently done by WJ B and HL D. The quality of evidence was subsequently classified into four categories (high, moderate, low, and very low) according to the corresponding evaluation criteria [26].

Results

**Figure 1:**
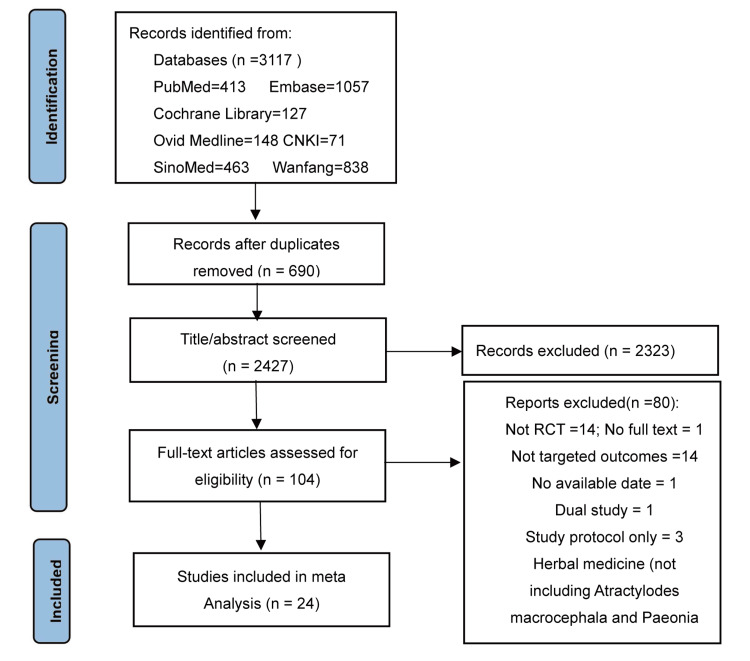
A flowchart showcasing the selection of studies Embase: Excerpta Medica database; MEDLINE: Medical Literature Analysis and Retrieval System Online; RCT: randomized controlled trial

We searched 3,117 article citations using the search strategy; of these, 104 published articles appeared to be relevant and were retrieved for further evaluation, of which, for various reasons, 80 were excluded, and finally 24 eligible articles with 3,768 participants met our inclusion criteria (Figure 1).

Risk of Bias Assessment

**Figure 2:**
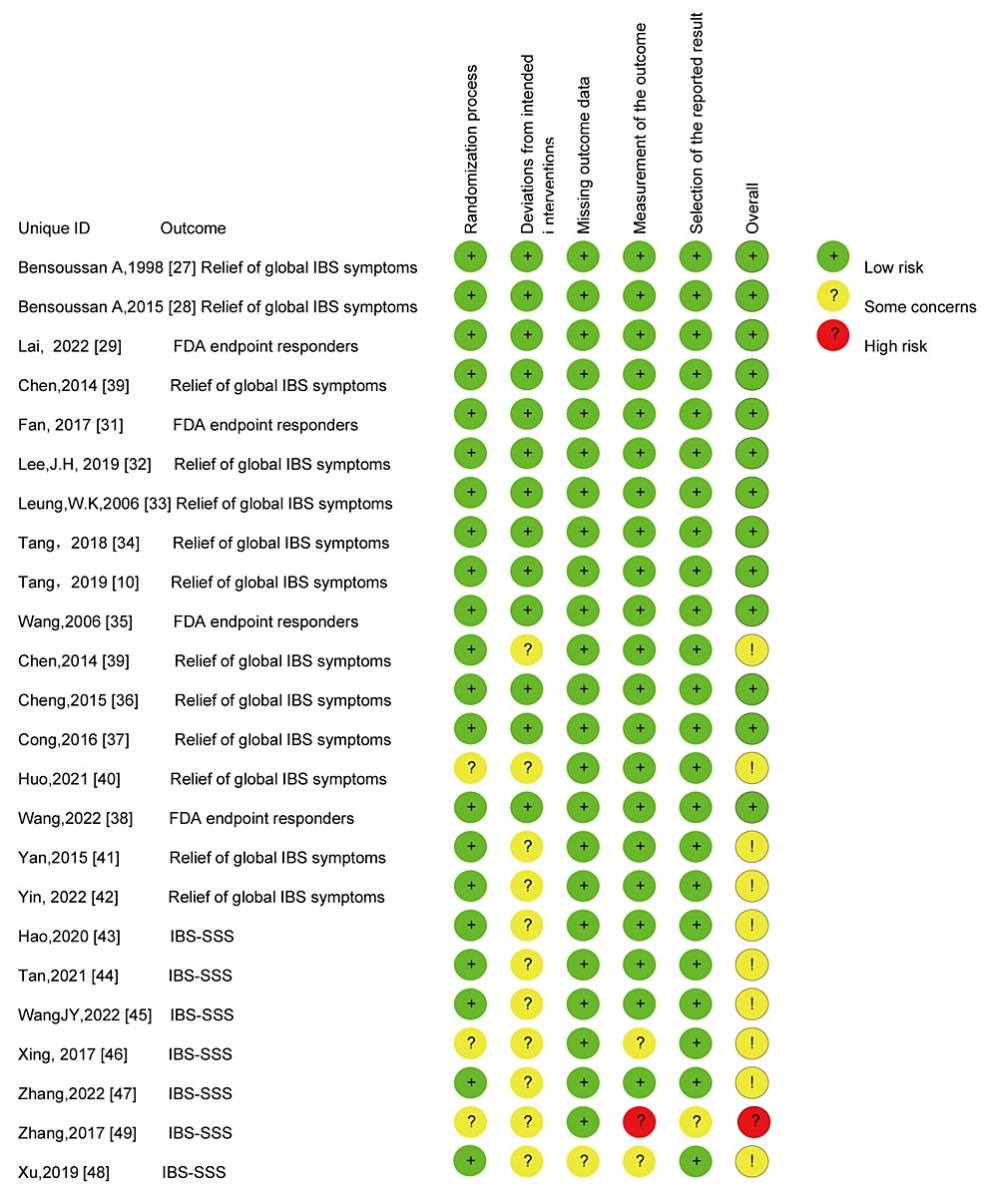
Risk of bias assessment of studies

**Figure 3:**
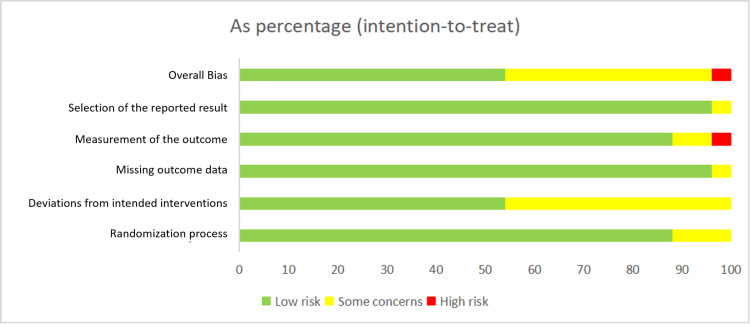
The bias assessment of all articles

Thirteen [27-38] of the 24 articles were at low risk of bias, 10 were of some concern [39-48], and one was at high risk of bias [49]. The bias assessment of all articles is shown in Figures 2-3.

The detailed characteristics of individual trials are shown in Table 5.

**Table 5:** Detailed characteristics of individual trials CHM: Chinese herbal medicine; IBS: irritable bowel syndrome; IBS-D: irritable bowel syndrome with diarrhea; IBS-C: irritable bowel syndrome with constipation; IBS-M: irritable bowel syndrome mixed type; TXYF: Tong Xie Yao Fang

Study	Design	Sample size	Mean age of CHM (year)	Female of CHM (%)	IBS subtype	Intervention	Control	Location	Diagnostic criteria	Treatment duration (weeks)
Tang, 2019 [10]	Multicenter	342	43.97	50.3	IBS-D	Tongxiening granules	Pinaverium	China	Rome III	6
Bensoussan A, 1998 [27]	Multicenter	116	47.6	0.61	IBS-C, IBS-D, and IBS-M	Standardized CHM	Placebo	Australia	Rome I	16
Bensoussan A, 2015 [28]	Multicenter	125	48.2	93.4	IBS-C	Standardized CHM	Placebo	Australia	Rome III	8
Lai, 2022 [29]	Multicenter	240	38.6	60	IBS-D	TXYF	Placebo	China	Rome IV	4
Chen, 2018 [30]	Multicenter	160	35.4	48.8	IBS-D	TXYF granules	Placebo	China	Rome III	4
Fan, 2017 [31]	Multicenter	1044	36.3	58	IBS-D	TXYF granules	Placebo or pinaverium	China	Rome III	4
Lee, 2019 [32]	Single	80	38.05	20	IBS-D	Samryungbaekchul-san	Placebo or otilonium bromide	Korea	Rome III	8
Leung, 2006 [33]	Single	119	45.4	48.3	IBS-D	TXYF	Placebo	China	Rome II	8
Tang, 2018 [34]	Multicenter	206	42.88	37.4	IBS-D	Chang'an Ⅰ recipe	Placebo	China	Rome III	8
Wang, 2006 [35]	Single	57	37.1	44.8	IBS-D	Tongxiening granules	Placebo	China	Rome II	3
Cheng, 2015 [36]	Single	62	NA	53.1	IBS-D	Chaishao tiaogan decoction	Trimebutine maleate dispersible tablets	China	Rome III	8
Cong, 2016 [37]	Single	120	43.9	52.5	IBS-D	Modified Changjitai (CJT)	Pinaverium	China	Rome III	8
Wang, 2022 [38]	Multicenter	168	36.5	44	IBS-D	TXYF	Pinaverium	China	Rome IV	4
Chen, 2014 [39]	Single	116	38.5	34.5	IBS-D	Yigan Fupi decoction	Pinaverium	China	Rome III	4
Huo, 2021 [40]	Single	96	31.2	56.3	IBS-D	TXYF	Pinaverium	China	Rome IV	4
Yan, 2015 [41]	Single	61	41.9	58.1	IBS-D	He Huan Ling Zhu Fang	Trimebutine maleate dispersible tablets	China	Rome III	8
Yin, 2022 [42]	Single	61	38.7	24.4	IBS-D	Lichang beverage	Bifico combination with Dicetel	China	Rome IV	4
Hao, 2020 [43]	Single	126	39	64.3	IBS-D	TXYF granules	Pinaverium	China	NA	8
Tan, 2021 [44]	Single	90	40.6	46.7	IBS-D	Qilian Jiechangning decoction	Trimebutine maleate capsules and bifidobacterium quadruple viable tablets	China	Rome IV	4
Wang, 2022 [45]	Single	80	40	37.5	IBS-D	Shenling Baizhu powder	Pinaverium	China	Rome IV	4
Xing, 2017 [46]	Single	88	45.5	36.4	IBS-D	Jianpi Huashi soup	Pinaverium	China	Rome III	4
Zhang, 2022 [47]	Single	68	45.9	47.1	IBS-D	Spleen and stomach health granules	Paroxetine hydrochloride and piviramine	China	Rome IV	4
Xu, 2019 [48]	Single	63	41.03	53.1	IBS-D	Guipi decoction	Pinaverium	China	Rome IV	4

Relief of Global IBS Symptoms

**Figure 4:**
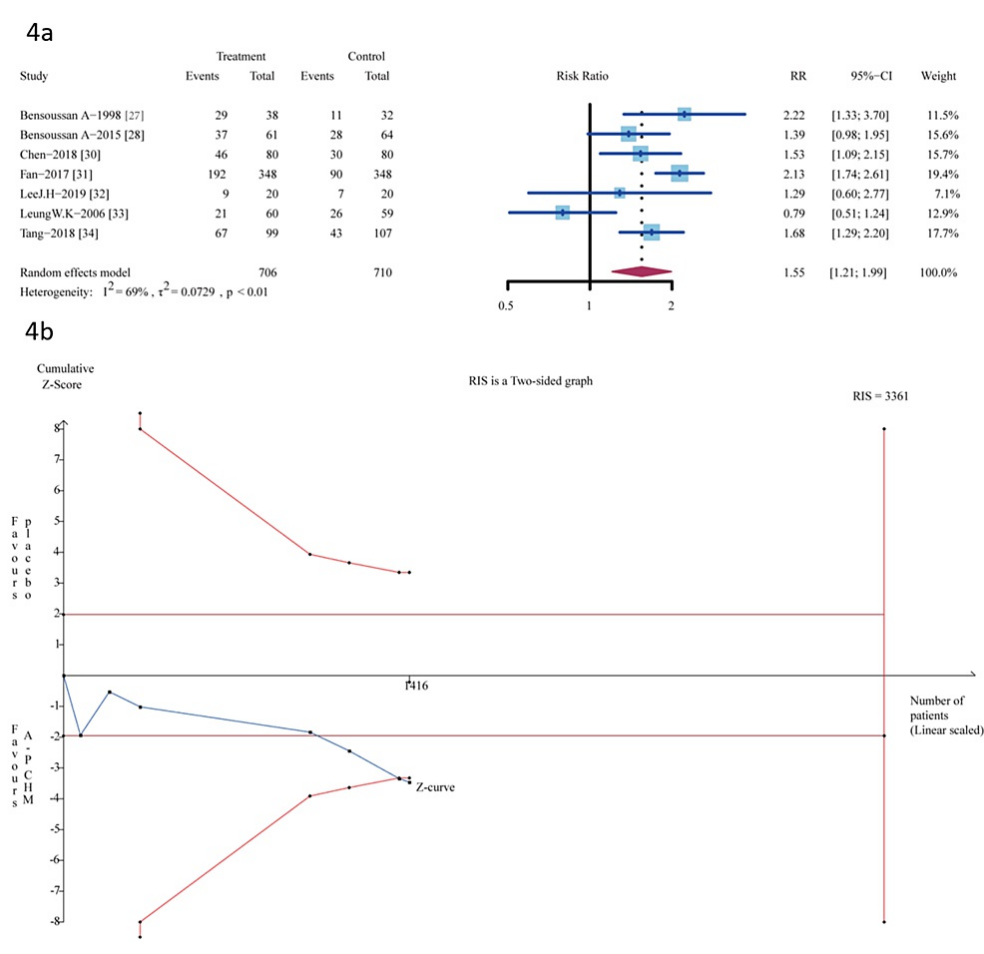
Meta-analysis and TSA analysis of A-P CHM vs. placebo in the proportion of patients who were “relief of global IBS symptoms” responders 4a: The results of the meta-analysis; 4b: The results of TSA: error α=5%, β=20%, incidence in the control arm =27%, RRR=30%. The red vertical line represented the RIS for the comparison, the blue line represented the cumulative Z curve, and the black dots represented the included studies. TSA: trial sequential analysis; A-P CHM: *Atractylodes macrocephala*-*Paeonia lactiflora *class formula; IBS: irritable bowel syndrome; RRR: relative risk reduction; RIS: required information size

Sixteen trials provided data for the outcome assessment of the proportion of patients who were “relief of global IBS symptoms” responders. Seven trials [27,28,30-34] were A-P CHM (n=706) vs. placebo (n=710); A-P CHM had significantly a higher proportion of global IBS symptom relief (RR 1.55 (95%CI, 1.21-1.99); I^2^ =69%; p < 0.01) NNT=6 (Figure 4a).

**Figure 5:**
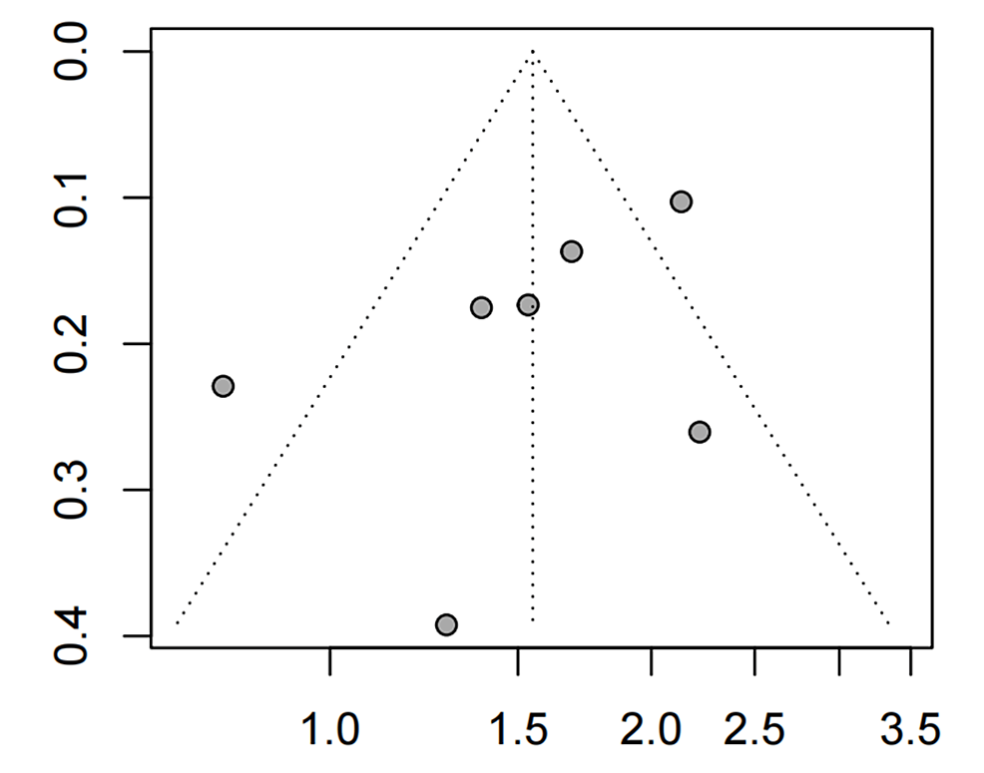
The funnel plot of global IBS symptom relief (A-P CHM vs. placebo) IBS: irritable bowel syndrome; A-P CHM: A*tractylodes macrocephala*-*Paeonia lactiflora* class formula

Based on the visual inspection of the funnel plot, we found no asymmetry (p=0.246, Figure 5).

**Figure 6:**
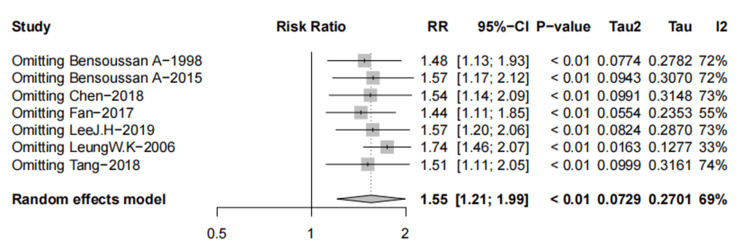
The sensitivity analysis of global IBS symptom relief (A-P CHM vs. placebo) IBS: irritable bowel syndrome; A-P CHM: *Atractylodes macrocephala-Paeonia lactiflora* class formula

Results from sensitivity analysis showed that the exclusion of any single study did not influence the overall estimate (Figure 6).

The evidence relating to A-P CHM and placebo for global IBS symptom relief was downgraded to low quality using the Grading of Recommendations Assessment, Development, and Evaluation (GRADE) protocol (Table 6).

**Table 6:** The overall certainty of evidence across RCTs CHM: Chinese herbal medicine; RCT: randomized controlled trial; IBS: irritable bowel syndrome; IBS-SSS: IBS severity scoring system; AR: adequate relief; RR: relative ratio; AE: adverse event; RoB: risk of bias

Author(s): Date: 2023-06-21 Question: Should CHM (baizhu baishao) vs. placebo or others be used for IBS? Settings: RCT Bibliography:													
Quality assessment							No of patients		Effect		Quality	Importance	
No of studies	Design	Risk of bias	Inconsistency	Indirectness	Imprecision	Other considerations	CHM (baizhu baishao)	Placebo or others	Relative (95% CI)	Absolute			
Relief of global IBS symptoms vs. placebo (follow-up 0-16 weeks; assessed with IBS-SSS or relief of global IBS symptoms or AR)													
7	Randomized trials	No serious risk of bias	Serious1	No serious indirectness	Serious2	None	401/706 (56.8%)	235/710 (33.1%)	RR 1.55 (1.21 to 1.99)	182 more per 1000 (from 70 more to 328 more)	LOW	CRITICAL	
								100%		550 more per 1000 (from 210 more to 990 more)			
								0%		-			
Abdominal pain (FDA) vs. placebo (follow-up 7-57 weeks; assessed with a decrease in the endpoint at least 30%)													
3	Randomized trials	No serious risk of bias	No serious inconsistency	No serious indirectness	No serious imprecision	None	273/497 (54.9%)	141/496 (28.4%)	RR 1.94 (1.65 to 2.27)	267 more per 1000 (from 185 more to 361 more)3	HIGH	CRITICAL	
								100%		940 more per 1000 (from 650 more to 1000 more)			
								0%		-			
Relief of global IBS symptoms vs. Western medication (follow-up 12-24 weeks; assessed with relief of global IBS symptoms)													
9	Randomized trials	Serious4	Serious5	No serious indirectness	No serious imprecision	None	599/826 (72.5%)	511/760 (67.2%)	RR 1.15 (1.03 to 1.29)	101 more per 1000 (from 20 more to 195 more)	LOW	CRITICAL	
								50%6		75 more per 1000 (from 15 more to 145 more)			
								50%6		75 more per 1000 (from 15 more to 145 more)			
Abdominal pain (FDA) vs. pinaverium (assessed with a decrease in the endpoint by at least 30%)													
2	Randomized trials	No serious risk of bias	Very serious7	No serious indirectness	Serious8	None	257/432 (59.5%)	290/432 (67.1%)	RR 0.96 (0.69 to 1.33)	27 fewer per 1000 (from 208 fewer to 222 more)	VERY LOW	IMPORTANT	
								100%3		40 fewer per 1000 (from 310 fewer to 330 more)			
								0%3		-			
Stool consistency (FDA) vs. placebo (follow-up 7-57 weeks; assessed with patients’ stool consistencies (became normal))													
3	Randomized trials	No serious risk of bias	Serious9	No serious indirectness	Serious2	None	368/497 (74%)	112/496 (22.6%)	RR 2.67 (1.64 to 4.37)	377 more per 1000 (from 145 more to 761 more)	LOW	CRITICAL	
								100%3		1000 more per 1000 (from 640 more to 1000 more)			
								0%3		-			
Stool consistency (FDA) vs. pinaverium (assessed with a decrease in the endpoint by at least 50% or patients’ stool consistencies became normal )													
2	Randomized trials	No serious risk of bias	Serious10	No serious indirectness	Serious2	None	340/432 (78.7%)	241/432 (55.8%)	RR 1.33 (1.06 to 1.47)	184 more per 1000 (from 33 more to 262 more)	LOW	IMPORTANT	
								100%3		330 more per 1000 (from 60 more to 470 more)			
								0%3		-			
IBS-SSS vs. placebo (follow-up 0-14 weeks; measured with a score; range of scores: 106-166; better indicated by lower values)													
2	Randomized trials	No serious risk of bias	No serious inconsistency	No serious indirectness	Serious11	None	112	95	-	SMD 0.71 lower (0 to 0 higher)12	MODERATE	IMPORTANT	
IBS-SSS vs. Western medication (follow-up 12-24 weeks; measured with a score; range of scores: 60-215; better indicated by lower values)													
14	Randomized trials	Very serious13	Very serious5	No serious indirectness	Serious2	None	648	581	-	SMD 1.19 lower (0 to 0 higher)14	VERY LOW	IMPORTANT	
AE VS. placebo (follow-up 0-24 weeks; assessed with AE%)													
5	Randomized trials	No serious risk of bias	Serious5	No serious indirectness	No serious imprecision	None	5/570 (0.88%)	63/569 (11.1%)	RR 1.33 (0.99 to 1.79)	37 more per 1000 (from 1 fewer to 87 more)	MODERATE	IMPORTANT	
								100%3		330 more per 1000 (from 10 fewer to 790 more)			
								0%3		-			
AE vs. pinaverium (follow-up 0-14 weeks; assessed with AE %)													
4	Randomized trials	Serious15	Serious5	No serious indirectness	No serious imprecision	None	80/597 (13.4%)	76/597 (12.7%)	RR 1.05 (0.79 to 1.4)	6 more per 1000 (from 27 fewer to 51 more)	LOW	IMPORTANT	
								75%3		37 more per 1000 (from 157 fewer to 300 more)			
								25%3		12 more per 1000 (from 52 fewer to 100 more)			
1 I 2 = 69%; 2 The confidence interval is wide; 3 Bias risk assessment based on Rob2; 4 The risk of bias in 4 studies is of some concern; 5 Forest map overlap is not good; 6 Results based on the risk of bias; 7 I 2 = 93% 8 Results based on the risk of bias; The confidence interval of forest map is wide; 9 I 2 = 88%; 10 I 2 = 85%; 11 The sample size is small; 12 -0.99 to -0.43; 13 There is some concern about the risk of bias in the 10 studies, high about the risk of bias in the 1 study; 14 -1.09,-0.6; 15 There is some concern about the risk of bias in one study													

The TSA analysis showed that according to the previous meta-analysis [50], which assumed 27% placebo in the proportion of relief of global IBS symptoms and relative risk reduction (RRR)=30%, the RIS required 3361 participants and the accrued sample size (n=1,416) of this meta-analysis. Figure 4b shows that the cumulative Z-curve crosses both the conventional and TSA bounds, indicating that although the cumulative amount of information does not reach the desired value, no more trials are needed to obtain a positive conclusion in advance.

**Figure 7:**
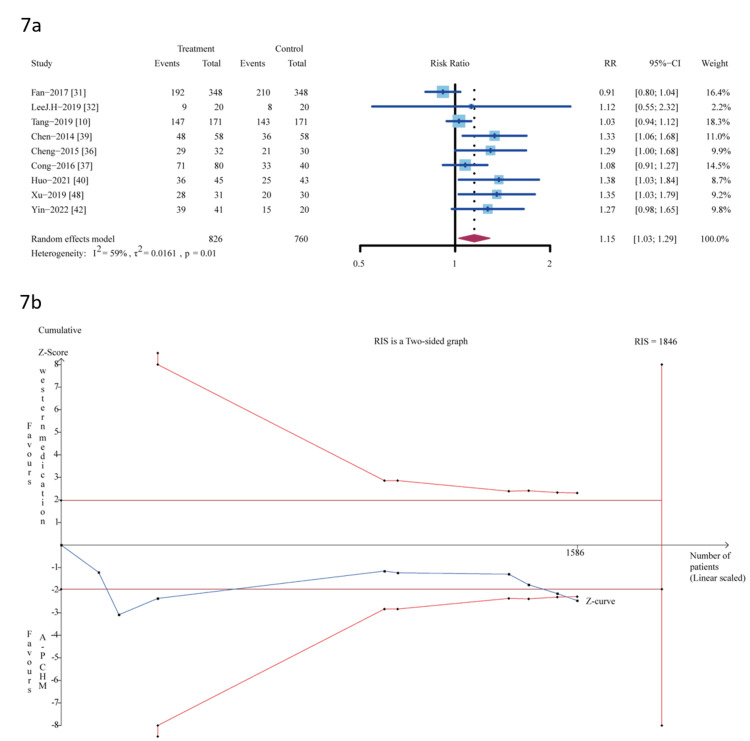
Meta-analysis and TSA analysis of A-P CHM vs. Western medication in the proportion of patients who were “relief of global IBS symptoms” responders 7a: The results of the meta-analysis; 7b: The results of TSA: error α=5%, β=20%, incidence in the control arm =60%, RRR = 20%. The red vertical line represents the RIS for the comparison, the blue line represents the cumulative Z curve, and the black dots represent the included studies. TSA: trial sequential analysis; A-P CHM: *Atractylodes macrocephala*-*Paeonia lactiflora* class formula; IBS: irritable bowel syndrome; RRR: relative risk reduction; RIS: required information size

Nine trials [10,31,32,36,37,39,40,42,48] were A-P CHM (n=826) vs. Western medication (n=760); A-P CHM had significantly a higher proportion of global IBS symptom relief (RR 1.15 (95%CI, 1.03-1.29); I^2^ =59%; p=0.01) NNT=14 (Figure 7a).

**Figure 8:**
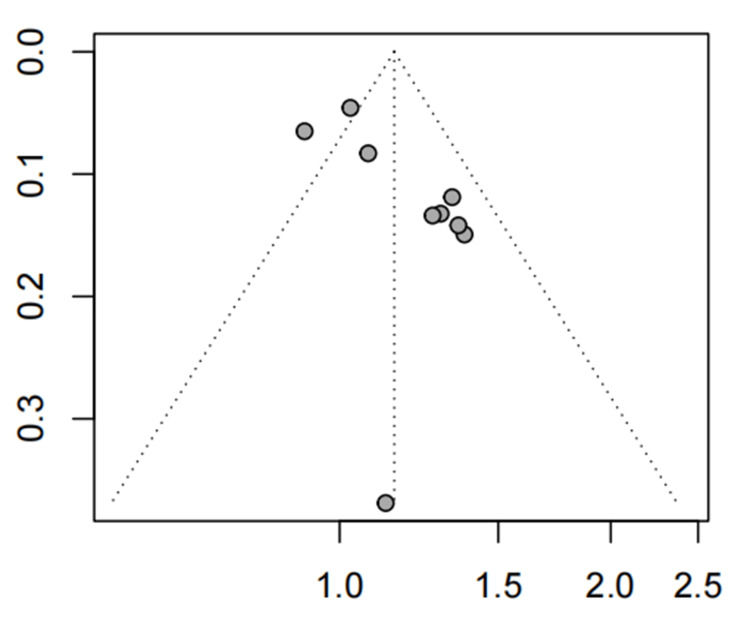
The funnel plot of global IBS symptom relief (A-P CHM vs. Western medication) IBS: irritable bowel syndrome; A-P CHM: *Atractylodes macrocephala-Paeonia lactiflora* class formula

Based on the visual inspection of the funnel plot, we found an asymmetry (p=0.0117, Figure 8).

**Figure 9:**
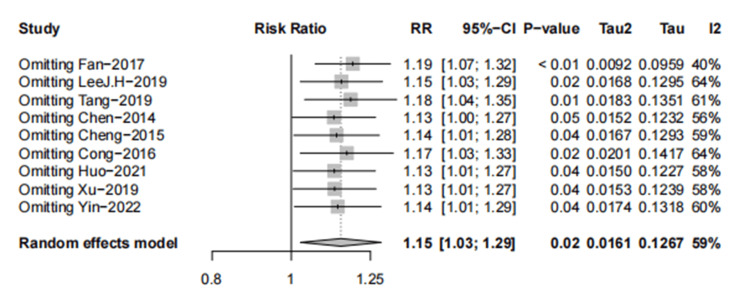
The sensitivity analysis of global IBS symptom relief (A-P CHM vs. Western medication) IBS: irritable bowel syndrome; A-P CHM: *Atractylodes macrocephala-Paeonia lactiflora* class formula

Results from sensitivity analysis showed that the exclusion of any single study did not influence the overall estimate (Figure 9).

The evidence relating to A-P CHM and Western medication for global IBS symptom relief was downgraded to low quality using the GRADE protocol (Table 6). The TSA analysis showed that assuming 60% Western medication [31] in the proportion of relief of global IBS symptoms and RRR=20%, the RIS required 1,846 participants and the accrued sample size (n=1586) of this meta-analysis. Figure 7b shows that the cumulative Z-curve crosses both the conventional and TSA bounds, indicating that although the cumulative amount of information does not reach the desired value, no more trials are needed to obtain a positive conclusion in advance.

The FDA Endpoint Responders

Only one trial [31] provided data for the outcome assessment of the proportion of patients who were composite FDA endpoint responders; there were 166 responders out of 348 in A-P CHM, placebo was 47/348, and pinaverium was 141/348.

**Figure 10:**
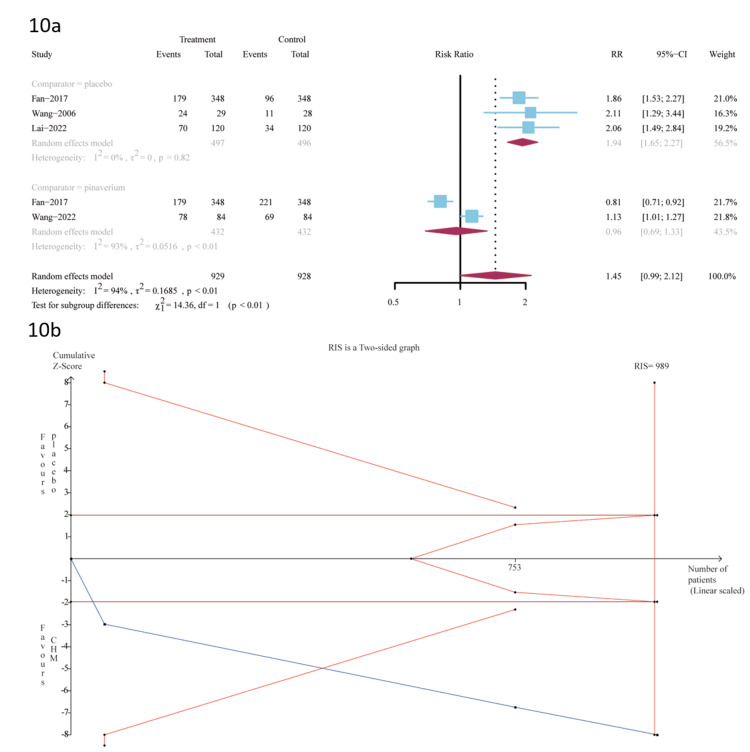
10a: FDA endpoint abdominal pain relief; 10b: The TSA analysis of FDA endpoint abdominal pain relief (A-P CHM vs. placebo) TSA: trial-sequential analysis; A-P CHM: *Atractylodes macrocephala-Paeonia lactiflora* class formula

Five trials provided data for the outcome assessment of the proportion of patients who were in the FDA-endpoint abdominal pain relief group. Three trials [29,31,35] were A-P CHM (n=497) vs. placebo (n=496); A-P CHM had significantly a higher proportion of FDA endpoint abdominal pain relief (RR 1.94 (95%CI, 1.65-2.27); I^2^ =0%; p = 0.82) NNT=3 (Figure 10a).

The evidence relating to A-P CHM and the placebo of FDA endpoint abdominal pain relief was of high quality using the GRADE protocol (Table 6). The TSA analysis showed that assuming 34% placebo [50] in the proportion of relief of FDA endpoint abdominal pain and RRR=24%, the RIS required 989 participants and the accrued sample size (n=993) of this meta-analysis. Figure 10b shows that the cumulative Z curve crossed both the conventional and TSA bounds, and the cumulative amount of information reached the desired value, indicating a statistically significant difference between A-P CHM and placebo. In the two trials [31, 38], A-P CHM (n=432) vs. pinaverium (n=432), there was no significant difference between the two groups (RR 0.96 (95% CI, 0.69-1.33); I2 =93%; p＜0.01) NNT=9 (Figure 10a). The evidence relating to A-P CHM and pinaverium of FDA endpoint abdominal pain relief was downgraded to very low quality using the GRADE protocol (Table 6).

**Figure 11:**
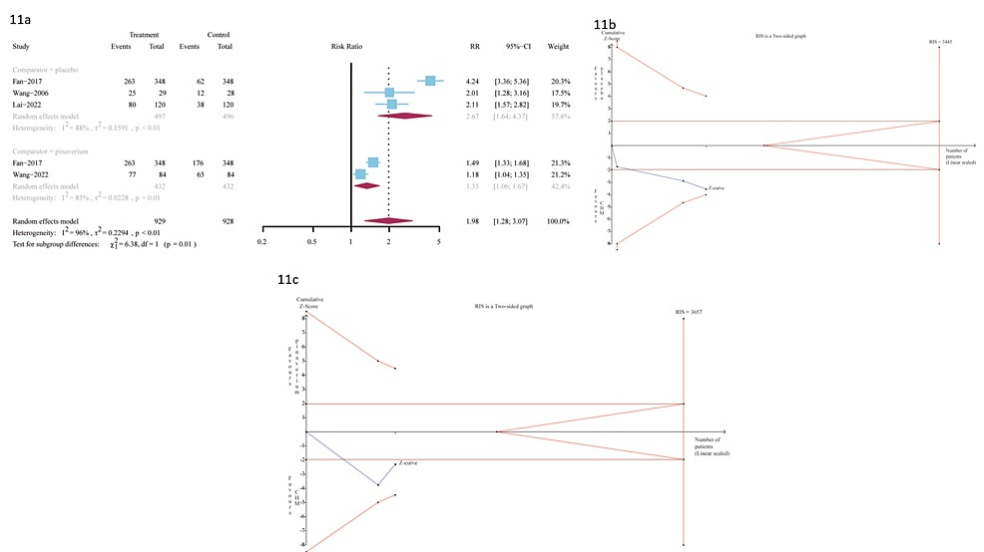
11a: FDA endpoint stool consistency relief; 11b: The TSA analysis of FDA endpoint stool consistency relief (A-P CHM vs. placebo); 11c: The TSA analysis of FDA stool consistency pain relief (A-P CHM vs. pinaverium) TSA: trial-sequential analysis; A-P CHM: *Atractylodes macrocephala-Paeonia lactiflora* class formula

Five trials provided data for the outcome assessment of the proportion of patients who received FDA endpoint stool consistency relief. Three trials [29,31,35] were A-P CHM (n=497) vs. placebo (n=496); A-P CHM had significantly a higher proportion of FDA endpoint stool consistency relief (RR 2.67 (95%CI, 1.64-4.37); I^2^ =88%; p <0.01) NNT=2 (Figure 11a).

The evidence relating to A-P CHM and placebo of FDA endpoint stool consistency relief was downgraded to low quality using the GRADE protocol (Table 6). The TSA analysis showed that assuming an 18% placebo in the proportion of relief of FDA endpoint stool consistency [50] and RRR=40%, the RIS required 3,445 participants and the accrued sample size (n=993) of this meta-analysis. Figure 11b shows that the cumulative Z curve crossed the traditional boundary value but did not cross the trial sequential boundaries, and the cumulative amount of information has fallen short of expectations, indicating A-P CHM was better than placebo for relief of FDA endpoint stool consistency and possible false positive results. Two trials [50] were A-P CHM (n=432) vs. pinaverium (n=432); A-P CHM had significantly a higher proportion of FDA endpoint stool consistency relief (RR 1.33 (95%CI, 1.06-1.67); I2 =85%; p=0.01] NNT=6 (Figure 11a). The evidence relating to A-P CHM and pinaverium of FDA endpoint stool consistency relief was downgraded to low quality using the GRADE protocol (Table 6). The TSA analysis showed that assuming 51% pinaverium in the proportion of relief of FDA endpoint stool consistency [31] and RRR=25%, the RIS required 3,657 participants and the accrued sample size (n=864) = 864 in this meta-analysis). Figure 11c shows that the cumulative Z curve crossed the traditional boundary value but did not cross the trial sequential boundaries, and the cumulative amount of information has fallen short of expectations, indicating A-P CHM was better than pinaverium in terms of relief of FDA endpoint stool consistency and possible false positive results.

The IBS-SSS Score

**Figure 12:**
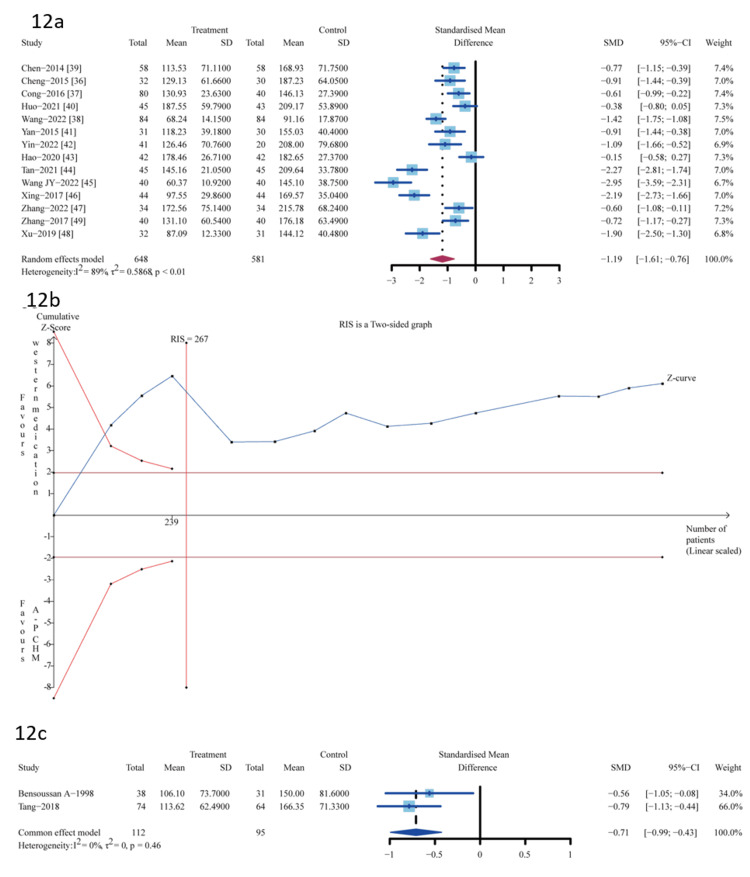
Meta-analysis and TSA analysis of the IBS-SSS score 12a: The results of the meta-analysis of A-P CHM vs. Western medication in the IBS-SSS score; 12b: The results of TSA of A-P CHM vs. Western medication in the IBS-SSS score: error α=5%, β=20%; 12c: The results of the meta-analysis of A-P CHM vs. placebo in the IBS-SSS score. The red vertical line represents the RIS for the comparison, the blue line represents the cumulative Z curve, and the black dots represent the included studies. TSA: trial sequential analysis; A-P CHM: *Atractylodes macrocephala*-*Paeonia lactiflora* class formula; IBS-SSS: irritable bowel syndrome-symptom severity score; RIS: required information size

Sixteen trials provided data for the outcome assessment of the IBS-SSS score. Fourteen trials [37-49] were A-P CHM (n=648) vs. Western medication (n=581); A-P CHM IBS-SSS score was lower than that of Western medication (SMD -1.19, 95% CI, -1.61--0.76); I^2^ =89%; p <0.01) (Figure 12a).

**Figure 13:**
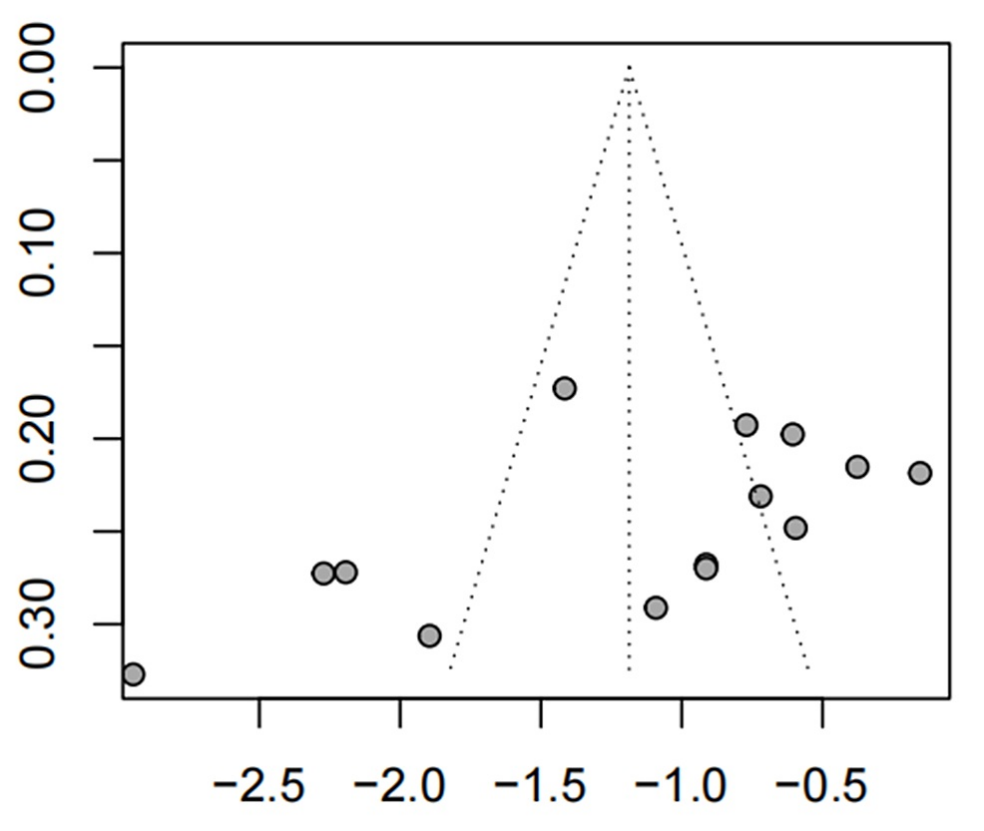
The funnel plot of IBS-SSS (A-P CHM vs. Western medication) IBS-SSS: IBS severity scoring system; A-P CHM: *Atractylodes macrocephala-Paeonia lactiflora* class formula

Based on the visual inspection of the funnel plot, we found no asymmetry (p=0.0622, Figure 13).

**Figure 14:**
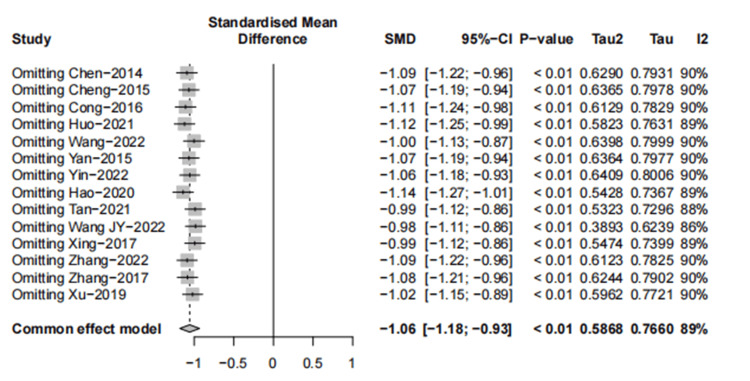
The sensitivity analysis of IBS-SSS (A-P CHM vs. Western medication) IBS-SSS: IBS severity scoring system; A-P CHM: *Atractylodes macrocephala-Paeonia lactiflora* class formula

Results from sensitivity analysis showed that the exclusion of any single study did not influence the overall estimate (Figure 14).

The evidence relating to A-P CHM and Western medication's IBS-SSS score was downgraded to very low quality using the GRADE protocol (Table 6). The TSA analysis showed that the RIS required 267 participants, and the accrued sample size (n=1229) of this meta-analysis. Figure 12b shows that the cumulative Z curve crossed trial sequential boundaries, and the cumulative amount of information has reached the desired value, indicating a statistically significant difference between A-P CHM and Western medication.

Two trials [27,34] were A-P CHM (n=112) vs. placebo (n=95); A-P CHM IBS-SSS score was lower than placebo (SMD -0.71, 95%CI, -0.99--0.43); I^2^ =0%; p=0.46) (Figure 12c). The evidence relating to A-P CHM and placebo of the IBS-SSS score was downgraded to moderate quality using the GRADE protocol (Table 6).

Adverse Events

**Figure 15:**
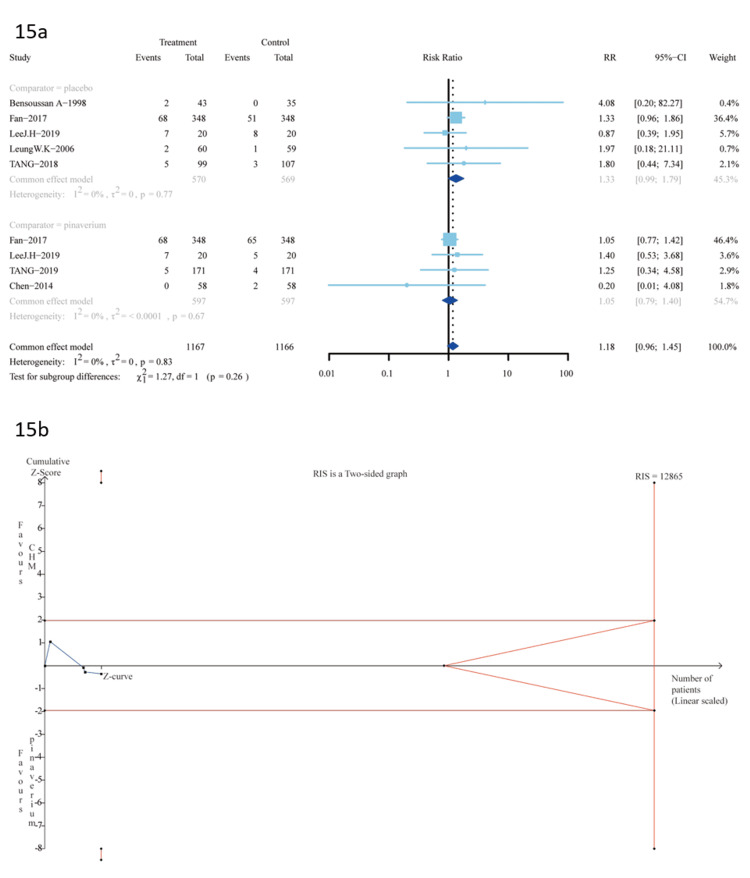
15a: adverse events; 15b: the TSA analysis of adverse events (A-P CHM vs. pinaverium) TSA: trial sequential analysis; A-P CHM: *Atractylodes macrocephala-Paeonia lactiflora* class formula

Nine trials provided data for the outcome assessment of adverse events. No serious adverse events happened. Five trials [27,31-34] were A-P CHM (n=570) vs. placebo (n=569); there was no significant difference between the two groups (RR 1.33 (95%CI, 0.99-1.79); I^2^=0%; p=0.77) NNH=33 (Figure 15).

The evidence relating to A-P CHM vs. placebo of adverse events was downgraded to moderate quality using the GRADE protocol (S5 Table).

Four trials [10,31,32,39] were A-P CHM (n=597) vs. pinaverium (n=597); there was no significant difference between the two groups (RR 1.05 (95%CI, 0.79-1.40); I2=0%; p=0.67) NNH=81 (Figure 15a). The evidence relating to A-P CHM vs. pinaverium of adverse events was downgraded to low quality using the GRADE protocol (Table 6). The TSA analysis showed that the RIS required 12,865 participants, and the accrued sample size (n=1194) of this meta-analysis. Figure 15b shows that the cumulative Z curve did not cross the trial sequential boundaries, and the cumulative amount of information did not reach the expected value, indicating that further studies are needed for validation.

Discussion

Our meta-analysis incorporated 24 studies. One study by Bensoussan et al. [27] focused on irritable bowel syndrome with constipation (IBS-C), irritable bowel syndrome with diarrhea (IBS-D), and irritable bowel syndrome mixed type (IBS-M); another study by Bensoussan et al. [28] specifically addressed IBS-C, while the remaining primarily catered to IBS-D. This leads us to conjecture that A-P CHM is more beneficial for the diarrhea-predominant type of irritable bowel syndrome.

Regarding the primary outcomes, A-P CHM demonstrated a significantly higher rate of global lBS symptom relief compared to either placebo or Western medication, with NNTs of 6 (vs. placebo) and 14 (vs. Western medication). These findings were further substantiated by TSA analysis; A-P CHM had significantly a higher proportion of FDA endpoint abdominal pain relief than placebo, with an NNT of 3. This was further corroborated by TSA analysis, indicating that our meta-analyses had an adequate sample size. The *Atractylodes macrocephala-Paeonia lactiflora* class formula had significantly a higher proportion of stool consistency relief than placebo or pinaverium; the NNT was 2 (vs. placebo) and 6 (vs. pinaverium), but the TSA analysis showed these possible false-positive results.

In relation to secondary outcomes, the lBS-SSS score for A-P CHM was lower than that for either Western medication or placebo at the conclusion of the treatment, a finding that was further validated by TSA analysis and confirmed the sufficient sample size of our meta-analysis. We also found that for adverse events, there was no significant difference between A-P CHM and placebo or Western medication; the NNH was 33 (vs. placebo) and 81 (vs. pinaverium); this was further confirmed by the TSA analysis.

Additionally, we utilized the GRADEprofiler to assess the overall certainty of evidence across RCTs. The evidence for FDA endpoint abdominal pain relief of A-P CHM and placebo was of high quality, while the evidence for IBS-SSS score and adverse events was of moderate quality. Evidence for other measures was downgraded to low or very low, indicating an overall low quality [51]. It was not until 2012 that the U.S. Food and Drug Administration recommended the use of endpoints assessing improvement in abdominal pain, improvement in bowel habits, or both in IBS-D. Since the publication of these recommendations, many RCTs of tightly designed drugs have followed these endpoints [52-54], although some have come close to these endpoints [55,56]. However, to the best of our knowledge, meta-analyses of herbal medicines for IBS have not used this endpoint. This information is important because it can inform sample size calculations for future high-quality RCTs. In addition, because the magnitude of the placebo response may affect the likelihood that a drug will show significantly better efficacy than a placebo, the choice of endpoints used to confirm efficacy may determine the success or failure of a drug. Consequently, we conducted a systematic review and meta-analysis of the efficacy of A-P CHM in treating irritable bowel syndrome, using the FDA-recommended endpoints as one of the outcome measures.

With 22 studies targeting IBS-D, it is reasonable to infer that A-P CHM may be more effective for diarrhea-predominant irritable bowel syndrome. According to Chinese medicine theory, IBS-D, despite its location in the intestines, is closely associated with the liver and rooted in the spleen. The liver is associated with the wood element, and the spleen is linked to the earth element. The record Jingyue quanquan notes: "Encountering anger leads to diarrhea... This involves diseases of both the liver and spleen, where the liver counters the earth element and the spleen receives Qi." In Medical Examination Volume II, it is stated that "The spleen is responsible for diarrhea, while the liver is responsible for pain. The liver deals with reality, and the spleen handles the virtual. Spleen deficiency leads to a solid liver, resulting in pain and diarrhea." Therefore, "pain" is primarily attributed to excessive wood strength, and "diarrhea" is linked to spleen deficiency and dampness.

In the context of diarrhea-type irritable bowel syndrome, the primary mechanism involves deficiencies in the liver and spleen, with wood overpowering the earth. The treatment approach involves addressing the imbalance by tonifying the earth element to counteract the wood element's excess [57].

*Atractylodes macrocephala*, with its bitter and sweet qualities, is warming and associated with the spleen and stomach meridians. It strengthens the spleen and alleviates diarrhea. *Paeonia lactiflora*, bitter and sour, slightly cold, pertains to the liver and spleen meridian. It softens the liver and relieves pain. The Chinese herbal formula, primarily composed of *Atractylodes macrocephala* and *Paeonia lactiflora*, aims to achieve therapeutic effects by addressing the interaction of wood and earth in the context of "diarrhea of wood in the earth." To the best of our knowledge, this study is the first to investigate the effectiveness of A-P CHM in the treatment of IBS.

The limitations of this systematic review and meta-analysis come from the studies available for synthesis. Nearly half of the trials were at high risk of bias or had some concerns, especially in trials published in Chinese journals, and the most common were deviations from intended interventions, especially the implementation of blinding, which suggests that attention should be paid to the design of blinding in future related studies to ensure the quality of the trials. And there is evidence of heterogeneity between the RCTs of A-P CHM and placebo and Western medicine treatments, and there is also evidence of publication bias between A-P CHM and Western medicines in improving the overall symptoms of IBS. This may lead to an overestimation of the treatment effect.

## Conclusions

Our study demonstrated the efficacy of A-P CHM in alleviating global IBS symptoms, improving FDA endpoint stool consistency, and reducing IBS-SSS scores. The findings of our meta-analysis were corroborated by the TSA analysis. Concurrently, no significant difference was observed in adverse events when compared with placebo or pinaverium, suggesting that A-P CHM might serve as a potential treatment for IBS patients, particularly those with IBS-D. This might provide a theoretical foundation for future optimization of herbal formulas for IBS-D. The overall certainty of the evidence was not high, underscoring the need for more rigorous RCTs in the future.
